# Strategies for Efficient Utilization of Corn Distillers Dried Grains with Solubles in Diets of Pigs: A Review

**DOI:** 10.3390/ani15121727

**Published:** 2025-06-11

**Authors:** Chunsheng Wang, Xinru Huang, Xue Liu, Ruixue Wang, Jianping Li, Qingwei Meng, Anshan Shan

**Affiliations:** College of Animal Science and Technology, Northeast Agricultural University, Harbin 150030, China; 15765129290@163.com (C.W.); s230501058@neau.edu.cn (X.H.); liuxue4320@163.com (X.L.); wrx1014405657@163.com (R.W.); ljpneau2018@163.com (J.L.)

**Keywords:** distillers dried grains with solubles (DDGS), pigs, efficient utilization, nutritional characteristics, strategies

## Abstract

With the increasing demand for ethanol fuel, corn distillers dried grains with solubles (DDGS) production is increasing annually. Current global ethanol output is approximately 120–130 billion liters annually, of which bioethanol constitutes 90–95% of total production. Corn DDGS is widely used as a feed ingredient for pigs due to its low cost and crude protein content ranging from 19% to 34%. However, corn DDGS is not effectively utilized because of factors such as raw material sources and inadequate drying processes, and a large portion of it is wasted and lost. It has become a difficult challenge to use DDGS in pig diets because of its weaknesses, including being rich in fiber and polyunsaturated fatty acids, which can lead to decreases in growth performance, feed conversion, and the pork shelf life of pigs. In recent years, researchers focusing on ways to improve the effective utility of corn DDGS in pig diets have made some progress. In this study, we review the nutritional characteristics of corn DDGS; the limiting factors in pig production, such as the higher concentration of non-starch polysaccharides, linoleic acid, and zein in corn DDGS; and the current improvement measures being taken to provide a reference for the improved utilization of corn DDGS resources in the diets of pigs.

## Simple Summary

As pork production grows globally, farmers and researchers seek affordable and sustainable feed options. While traditional protein sources like soybean meal or fishmeal are effective, they can be costly. This has led to increased interest in alternatives like corn distillers dried grains with solubles (DDGS), which is a low-cost, protein-rich by-product of ethanol production. However, DDGS is not perfect; its high fiber content and specific fats can soften pork fat and lower meat quality, which limits its use in pig diets. To address these issues, scientists are testing simple solutions, such as blending DDGS with common fats or oils like tallow, cottonseed oil, or soybean oil. These additives help balance the fat composition in pig feed, improving growth rates and meat quality. For example, adding small amounts of tallow or vegetable oils to DDGS-based diets has shown promise in enhancing pigs’ growth and keeping pork firm. Similarly, by-products like crude glycerol from biodiesel production are being explored to refine DDGS-based feeds further. While challenges remain—such as inconsistent results with certain additives—these innovations highlight a practical path toward making DDGS a more reliable and efficient feed resource. By combining cost-effective solutions with ongoing research, the pork industry can reduce waste, lower costs, and sustainably meet rising demand.

## 1. Introduction

With the growing number of people worldwide and the increasing industrialization of countries, the consumption of energy is steadily rising [1]. The global energy matrix remains critically dependent on non-renewable fossil hydrocarbons, such as petroleum and coal, whose accelerated exploitation drives resource depletion while generating carbon-intensive emissions that exacerbate anthropogenic climate destabilization [2]. Now, there is a shift in global fossil fuel consumption, and the world is finding renewable sources of energy. And ethanol fuel, a clean and renewable energy source, has natural advantages over fossil fuels [3]. Current global ethanol output is approximately 120–130 billion liters annually, of which bioethanol constitutes 90–95% of total production. Notably, the Renewable Fuel Association metrics indicate 112 billion liters of bioethanol was manufactured globally in 2023 [4]. Brazil exemplifies this growth trajectory: corn ethanol production escalated from 40 million liters in 2013 to 4.43 billion liters in 2024 through the synergistic integration of expanded cultivation zones, precision agricultural technologies, optimized agronomic protocols, and advanced biorefinery operations [5]. With every gallon of ethanol produced, approximately 2.6 kg of DDGS is produced [6]. Concurrently, U.S. corn-based ethanol facilities generated 22.6 million metric tons of corn DDGS in 2019 [7]. Corn distillers dried grains with solubles (DDGS), a by-product of fuel ethanol production, yields approximately 0.95 tons per 3 tons of corn processed. Its crude protein content of 19–34% is significantly lower than that of soybean meal, with 46–47% crude protein, yet its cost-effectiveness makes it a viable partial substitute for soybean meal and corn in pig diets. The prime objective of this review is to evaluate the potential of DDGS as a protein source in alleviating feed resource shortages, focusing on its comparative nutritional value and substitution strategies [8,9,10].

Nevertheless, DDGS contains a notably higher concentration of non-starch polysaccharides (NSPs) compared to corn [11]. While NSP can undergo partial fermentation in the intestinal tracts of monogastric animals, its energy is inefficiently utilized by pigs and poultry [12,13]. Moreover, as an antinutritional factor, NSP detrimentally impacts the digestibility of other nutrients, particularly in young animals [14]. For instance, a study by Bloxham et al. [15] found that feeding 30% DDGS reduced the gross energy digestibility of weaned pigs. To address the challenges posed by the high content of NSPs in DDGS and its restricted inclusion levels in pig diets, effective strategies include the addition of exogenous enzymes [16], fermentation [17], and pretreatment [18,19,20,21].

Dietary fatty acids directly affect the fatty acid profile of animal tissues, influencing pork’s nutritional quality [22]. DDGS, rich in polyunsaturated fatty acids (PUFAs) such as linoleic acid, leads to PUFA accumulation in pig adipose tissue when included at high levels [23]. This alters carcass traits, abdominal fat consistency, lipid composition, and pork shelf life [24,25]. To counteract elevated unsaturated fatty acids from DDGS and improve dietary feasibility, strategies include metabolic interventions and supplementation with vegetable/animal oils to balance fatty acid ratios [26,27]. A further limitation of DDGS is its high zein content, which is low in Lys and Trp but abundant in Leu [28,29]. Leu, along with Val and Ile, form the branched-chain amino acids (BCAAs), which share common catabolic pathways [30]. Excess BCAA intake accelerates their collective degradation, ultimately depleting essential amino acids [31]. While previous reviews and meta-analyses have broadly addressed the utilization of DDGS or focused on growth performance in pigs [7,32], this review discusses the limitation factors based on the nutritional characteristics of corn DDGS and uniquely integrates multiple strategies—including exogenous enzymes, pretreatment methods, metabolic regulation, fatty acid balancing, and amino acid optimization—to holistically address the nutritional limitations of DDGS.

## 2. Nutrient Composition, and Feeding Value

### 2.1. Carbohydrates

The NSP composition of corn DDGS mirrors that of corn, primarily comprising arabinoxylan (48.6%), cellulose (21.6%), and other hemicelluloses (29.8%) [33]. During DDGS production, corn starch is fermented into ethanol and carbon dioxide [34], concentrating residual nutrients by 2–3 times and positioning DDGS as a key source of energy, protein, fat, and minerals [35]. However, its nutritional profile varies significantly due to differences in raw corn quality, soluble matter content in pre-dried distillers dried grains (DDG), starch-to-ethanol conversion efficiency, and processing conditions, such as temperature and drying duration [36,37]. DDG, the post-fermentation residue after ethanol removal [38], exhibits compositional variability that directly impacts DDGS market value, nutrient digestibility, and total digestible nutrients [35]. DDGS composition critically influences its market value by affecting nutrient stability, digestibility, and feeding efficacy [35]. Nutritional variability across sources and production years is well-documented [35,39,40], exemplified by Olukosi and Adebiyi [41], who reported significant nutrient content fluctuations in DDGS samples (Table 1). For example, crude fiber (CF) was 6.2–11.3%, aid detergent fiber (ADF) was 8.6–18.5%, and neutral detergent fiber (NDF) was 27.7–51.0%. The coefficients of variation (CVs) for CF, ADF, and NDF were 15.1%, 24.2%, and 15.7%, respectively, and the means for CF, ADF, and NDF were 7.4%, 13.6%, and 36.6%, respectively. Cromwell et al. [39] reported that the means of DDGS for dry matter (DM), total carbohydrates, ADF, and NDF were 90.5%, 54.3%, 15.9%, and 38.8%, respectively, whereas the DM ranged from 87.1% to 92.7%, ADF from 11.4% to 20.8%, and NDF from 33.1% to 43.9%. These values are not substantially different from those reported by Olukosi and Adebiyi [41]. In the study by Belyea et al. [42], the NDF exhibited greater variability compared to other experiments. Whereas, Spiehs et al. [43] found that the mean for the DM, total carbohydrates, CF, and ADF were not different from those reported by Cromwell et al. [39]. 

### 2.2. Crude Protein and Amino Acid Content

**Figure 1:**
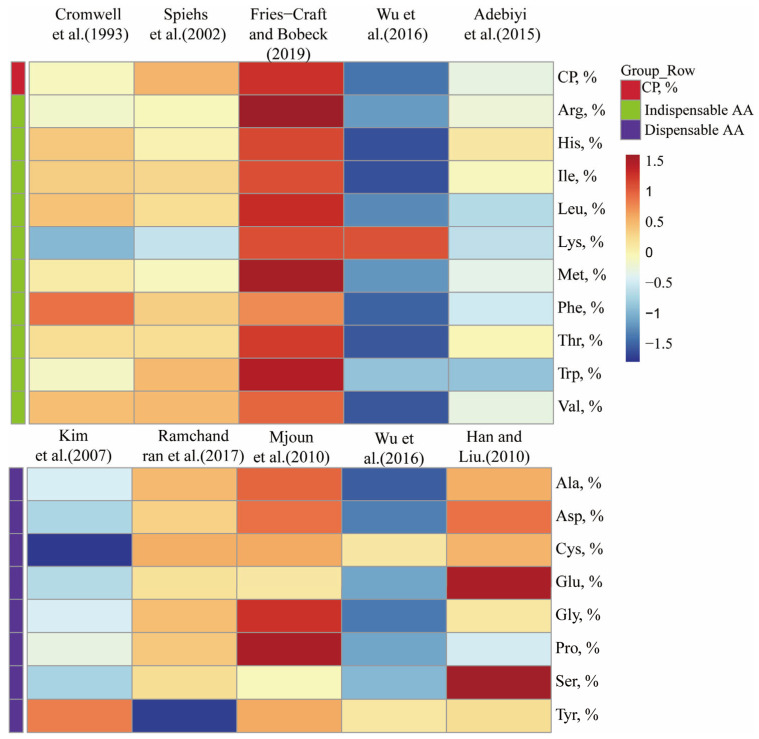
Indispensable and dispensable amino acid content in corn distillers dried grain with solubles (DDGS). AA, amino acid; CP, crude protein; Cromwell et al. [39]; Spiehs et al. [43]; Fries-Craft and Bobeck [46]; Adebiyi et al. [47]; Wu et al. [45]; Kim et al. [48]; Ramchandran et al. [49], Mjoun et al. [51], Wu et al. [45], and Han and Liu [50].

Corn DDGS contains a crude protein (CP) content of 19.8–34.1%, of which 40% is zein [44]. Zein is deficient in Lys and Trp but has high levels of Leu; therefore, soybean meal is commonly used in diets to supplement the Lys and Trp content [29]. The content means of Leu, Lys, and Trp in DDGS, were 3.41%, 0.96%, and 0.23%, respectively; the other amino acid (AA) content means of Arg, His, Ile, Met, Phe, Thr, and Val were 1.21%, 0.76%, 1.07%, 0.56%, 1.39%, 1.09%, and 1.40%, respectively, as shown in Figure 1. Spiehs et al. [43] and Wu et al. [45] observed that most essential amino acids were similar to the findings of Cromwell et al. [39], except for Lys, which exhibited significant variability. In the study of Fries-Craft and Bobeck [46], it was found that the content of Arg, Leu, Met, Thr, and Trp was higher compared with the other studies presented in Table 2. However, Adebiyi et al. [47] reported that most essential amino acids were lower in concentration compared to other studies, except for Lys. Kim et al. [48] reported that the mean values of Ala, Asp, Glu, Gly, Pro, Ser, and Tyr were 1.90%, 1.70%, 3.30%, 1.10%, 2.00%, 1.20%, and 1.20%, respectively. Ramchandran et al. [49] found that the mean values of Ala, Asp, Cys, Glu, Gly, Pro, and Ser were 2.08%, 1.89%, 0.59%, 4.14%, 1.20%, 2.26%, and 1.30%, respectively. In the other experiments, the amount of each nonessential amino acid was more variable; the reason for the differences in amino acid content may be due to differences in the corn feedstock and origin [45,50,51].

### 2.3. Ether Extract and Fatty Acid Composition

The ether extract (EE) content of DDGS ranged from 5.36% to 10.7%, as summarized from four studies in Table 2. In the studies of these individuals [52,53,54,55], it was reported that the means of total PUFAs, C18:2n-6, and C18:3n-3 were 55.66%, 52.97%, and 2.11%, respectively, indicating DDGS is rich in PUFAs, especially C18:2n-6. Additionally, the means of the total saturated fatty acids (SFAs), total monounsaturated fatty acids (MUFAs), C14:0, C16:0, C16:1, C18:0, C20:0, and C18:1n-9 were 15.99%, 24.77%, 0.20%, 12.23%, 0.16%, 2.59%, and 23.97%, respectively, as shown in Table 3. Notably, among these fatty acids, as the proportion of linoleic acid in the fatty acids increases, the overall unsaturation of the fat increases, which led to the hardness of pig fat decreasing [56]. Elevated levels of C18:2n-6 have been demonstrated to compromise bacon quality and introduce processing challenges [57]. In addition, this heightened unsaturation accelerates the oxidation process in pork, consequently diminishing its shelf life [23].

### 2.4. Energy

The way that energy is distributed is influenced by the physicochemical properties of the dietary ingredients and the physiological state of the pig, which affect the energy value of the feed ingredients and the energy requirements of the pig, respectively [58]. Digestible energy (DE) is the difference between the GE and feces energy; DE minus urinary energy and gaseous products of DE equals the metabolizable energy (ME); and DE minus heat increment equals the net energy (NE), which is further divided into maintenance NE and production NE [59]. As illustrated in Table 3, DDGS has a rich fat content and exhibits a higher GE compared to corn. However, the DE, ME, and NE of DDGS were lower than those of corn. This may be due to the higher CP content of DDGS during the production process, which might reduce the DE, ME, and NE values of DDGS [60]. In livestock production, the DE and ME systems may overestimate the energy value of high fiber diets such as DDGS [61,62]. In contrast, the NE system better represents the energy requirements of pigs fed DDGS than the DE and ME systems [62]. Therefore, the NE system is increasingly being adopted to promote more efficient use of DDGS in pig feed formulations.

**Table 3:** Concentration of energy in corn distillers dried grains with solubles (DDGS) fed to pigs.

Item	Corn	DDGS			
		Mean	SEM	Max	Min
GE, kcal/kg	4511	4527.8	215.48	5330	4142
DE, kcal/kg	3963	3556.5	113.87	3896	3408
ME, kcal/kg	3852	3406.5	131.76	3705	3157
NE, kcal/kg	3053	2292.2	156.43	2748	1924

Max, maximum; Min, minimum; SEM, standard error of the mean; GE, gross energy; DE, digestible energy, ME, metabolizable energy; and NE, net energy. Compiled from [45,63,64,65,66].

### 2.5. Amino Acid Digestibility

DDGS could replace soybean meal, corn, and other ingredients in diets of pigs [67,68]. Therefore, evaluating the digestible amino acid content is important for the application of DDGS in diets. True digestibility is more accurate than apparent digestibility; however, the calibrated determination of endogenous amino acids is complex. Therefore, the use of the standardized ileal digestible (SID) correction factor to adjust endogenous amino acids has been widely adopted in recent years.

Apparent ileal digestibility (AID) and SID of amino acids in DDGS have been extensively assessed and are detailed in Table 4. Notably, variations were observed in the digestibility of amino acids within DDGS samples, even when sourced from the same cereal grain [69,70]. Lys, in particular, exhibited greater variability in AID compared to other essential amino acids [69,70,71]. This disparity in Lys digestibility, when compared with other amino acids, is believed to stem from the heat-induced Maillard reactions of certain DDGS sources [72]. However, excluding Lys, the variations observed across the different DDGS sources remain consistent with typical fluctuations observed in other feed components. Interestingly, Stein et al. [61] found that DDGS samples with diminished Lys digestibility also tend to exhibit reduced Lys concentrations. The AID of Pro was more variable compared to other non-essential amino acids; this may be related to the DDGS fat content, because in the study of Shelby et al., the AID of Pro in pigs decreased as the fat content of the DDGS fed decreased [73]. Pig diets are a combination of feed ingredients to meet the nutritional requirements of pigs [74]. However, the utilization of nutrients in the different components is not consistent, and some nutrients are not utilized by the animal and are excreted via feces [75]. Therefore, it is important to estimate the proportion of each nutrient in each component of the feed that will be absorbed by the pig so that the dietary nutrients are consistent with the pig’s requirements [76]. With SID correction, Lys still showed high variability compared to the other essential amino acids, and Pro also exhibited high variability compared to other non-essential amino acids. However, these differences all tend to decrease, revealing that endogenous amino acids have some effect on the measurement of AID.

These findings highlight the significant variations in nutrient concentrations within DDGS based on their source. Moreover, these compositions may not always align with the standard reference values. Factors that contribute to these nutrient disparities include variations in original seed composition, starch fermentation efficiency, ethanol fermentation scales, soluble addition, and the drying process [21,40].

### 2.6. Carcass Characteristics

The inclusion of corn DDGS in pig diets has been widely studied due to its economic and nutritional benefits. However, its impact on carcass characteristics remains a critical consideration for pork producers. This analysis synthesizes findings from multiple studies in Table 5 to evaluate the effects of DDGS on key carcass characteristic.

The inconsistent HCW responses observed across 21 studies evaluating corn DDGS in pig diets—with 10 reporting reductions and 11 showing no significant changes—likely stem from variations in dietary formulation, DDGS compositional heterogeneity, and experimental design. Reductions in HCW are often attributed to increased dietary fiber content in DDGS, which promotes visceral organ hypertrophy and gastrointestinal tract proliferation, elevating maintenance energy demands and reducing nutrients available for carcass deposition [24,83,84]. Conversely, studies reporting no HCW differences often utilized precision-formulated diets balancing amino acids and energy, or employed DDGS with higher residual oil, mitigating energy deficits [25,83,84,85]. The inconsistent effects of DDGS on dressing percentage across 15 studies—with 4 reporting reductions and 11 showing no effect—may arise from variations in DDGS composition, dietary formulation strategies, and experimental protocols. Reductions in dressing percentage are often linked to high-fiber DDGS, which increases gastrointestinal tract mass and digesta volume, diverting nutrients to visceral organ hypertrophy rather than carcass deposition [24,84,86]. Studies observing no effects typically utilized DDGS with lower fiber content or balanced diets with supplemental lipids to maintain energy density, offsetting the fiber-induced energy dilution [25,61,85]. The stability of lean meat percentage across seven of eight experiments evaluating DDGS inclusion, with only one study reporting a decline, likely reflects compensatory dietary adjustments and inherent variability in DDGS nutrient utilization. Most studies maintaining lean percentage formulated diets with balanced standardized ileal digestible (SID) lysine to metabolizable energy ratios, ensuring sufficient amino acids for protein synthesis despite DDGS induced energy dilution [25,61,85]. The high residual oil content (7–10%) in some DDGS sources provided additional energy to support lean deposition, offsetting fiber-related energy losses [83,84]. The observed areas of the LM and loin eye across studies have not changed, which highlights the nuanced balance between dietary formulation, nutrient utilization, and methodological precision in DDGS-fed pig systems. The stability of these metrics in most trials likely stems from adequate maintenance of digestible amino acid profiles, particularly lysine and methionine, which supported sustained myofibrillar protein synthesis [25,61]. The observed increases in the iodine value (IV) of pork fat from pigs fed corn DDGS across 9 of 12 experiments are primarily driven by the high concentration of unsaturated fatty acids, particularly linoleic acid, in DDGS, which is deposited directly into adipose tissue due to dietary lipid absorption and reduced de novo fatty acid synthesis [83,87]. The residual oil in DDGS contains over 50% linoleic acid, which elevates the proportion of polyunsaturated fatty acids in fat depots, leading to higher IV as a measure of unsaturation [24]. These findings highlight the importance of tailored formulation strategies—such as oil content optimization, phase-specific inclusion, and lipid balancing—to mitigate variability in carcass outcomes while leveraging DDGS as a cost-effective feed ingredient.

**Table 5:** Effects of including corn distillers dried grains with solubles (DDGS) in diets fed to carcass characteristic of pigs.

Item	*n*	Response to Dietary Corn DDGS, No. of Experiments		
		Increased	Reduced	Not Changed
Hot carcass weight	21		10	11
Chilled carcass	1		1	
Chilling loss	2			2
Dressing	15		4	11
Lean meat	8		1	7
LM area	7			7
Loin eye area	4			4
LM depth	1			1
Backfat depth	11			11
Iodine value	12	9		3

Compiled from [24,25,71,83,84,85,88,89,90,91,92,93,94,95,96,97,98,99,100,101,102,103].

## 3. Strategies for the Efficient Utilization of Corn DDGS in Pig Diets

The limitations of corn DDGS in pig production arise from its inherent deficiencies. This review addresses these limitations by integrating exogenous enzymes, preprocessing, metabolic regulation, balancing fatty acids in the diet via adding oils of vegetable and animal origin, adjusting amino acid content, and enhancing the overall utility of corn DDGS in diets of pigs.

### 3.1. Exogenous Enzymes

NSPs, commonly referred to as fiber, are carbohydrate polymers with ten or more monomeric units that are neither hydrolyzed by endogenous enzymes nor absorbed in the small intestine [104]. Pigs lack the requisite endogenous carbohydrate enzymes and depend on microbial fermentation for the utilization of NSP [105]. Elevated levels of insoluble NSP in diets are typically viewed as antinutritional, as they can impede the digestibility of other nutrients and energy, thereby negatively influencing pig performance and decreasing carcass yield [106]. Nevertheless, introducing exogenous carbohydrates can mitigate some of these antinutritional effects.

The main NSPs in corn DDGS are arabinoxylan and galactomannan [107]. These NSP have been shown to reduce growth performance and inhibit energy and nutrient digestibility in pigs [108]. Therefore, it is beneficial to add enzymes to pig diets that are supplemented with high levels of DDGS. The addition of β-1,4-mannanase can directly target β-1,4-mannan in the feed ingredients [107]. Among the many different enzymes that target xylan, endo-1,4-β-xylanases are the most important, as they are directly involved in the cleavage of glycosidic bonds and the release of short xylooligosaccharides [109]. Xylooligosaccharides can be used as potential prebiotics [110]. O’Shea et al. [111] added 200 mg/kg xylanase to diets containing DDGS, which reduced the fecal odor emissions of the pigs but had no effect on the growth performance of the pigs compared to those fed DDGS alone (Table 6). However, Chen et al. [112] found that adding 1500 EPU/kg xylanase to diets containing DDGS increased the body weight (BW), average daily gain (ADG), and AID of the GE and NDF of the pigs compared to those fed DDGS alone. Pigs fed DDGS with high levels of NSPs resulted in increased digestive viscosity, which reduced feed digestibility and animal growth performance [113], whereas the addition of xylanase to diets containing DDGS decreased the jejunal digestive viscosity of the pigs relative to those fed DDGS alone [112]. Moreover, Yoon et al. [114] reported that as the concentration of mannanase in diets containing DDGS increased, the ADG and blood glucose of the pigs also increased linearly compared to those fed DDGS alone, and the apparent total tract digestibility (ATTD) of DM and CP of the pigs exhibited an improving trend compared to those fed DDGS alone.

Phosphorus (P) concentrations in DDGS were 0.49–0.61%, which was higher than the 0.38% of corn [72]. The addition of phytase to pig diets containing DDGS improved P utilization through the effective digestion of phytate antinutritional factors in the feedstuffs [115]. The effect obtained by the single enzyme addition was significant, but in the experiment by Lee et al. [116], the addition of mannanase and phytase to diets containing DDGS only improved the average daily feed intake (ADFI) of the pigs compared to those fed DDGS alone. Moreover, in the experiment of O’Shea et al. [111], the addition of protease to diets containing DDGS increased the AID of the GE in the pigs relative to those fed DDGS alone, but the simultaneous addition of 200 mg/kg protease and 200 mg/kg xylanase to the diets containing DDGS did not affect the pigs compared to those fed DDGS alone. In the experiment by Agyekum et al. [16], it was found that the simultaneous addition of 4000 U/kg xylanase and 300 U/kg β-glucanase to the diets containing DDGS did not affect the growth performance of the pigs compared to those fed DDGS alone. In summary, adding a complex enzyme to diets containing DDGS is not as beneficial as adding a single enzyme.

**Table 6:** The effect of adding exogenous enzymes on pigs fed corn distillers dried grains with solubles (DDGS).

Animal	Improvement Mode	DDGS Levels	Experiment Duration	Main Effects	Reference
Pigs 22.4 ± 0.7 kg	4000 U/kg xylanase and 300 U/kg β-glucanase mixture	30%	21-day feeding	Increased MCT1 mRNA expression in the ileum of the pigs relative to feeding DDGS alone	[16]
Pigs 30 kg	0.2 g/kg compound enzymes	10, 15%	Final BW 100 kg	Increased C18:2 and C20:4 in the longissimus muscle of the pigs	[117]
Pigs 34.2 ± 2.1 kg	200 mg/kg protease	30%	28-day feeding	Increased growth performance and AID of the GE in the pigs	[111]
Pigs 34.2 ± 2.1 kg	200 mg/kg xylanase	30%	28-day feeding	Decreased odor from the manure emissions of the pigs	[111]
Pigs 34.2 ± 2.1 kg	200 mg/kg protease and 200 mg/kg xylanase	30%	28-day feeding	No beneficial effects on the pigs	[111]
Pigs 63.92 ± 1.50 kg	0.14% enzyme premix (mannanase + phytase)	10, 20%	Final BW 107 ± 4 kg	Increased feed conversion ratio	[116]
Pigs 10.7 ± 1.2 kg	1500 EPU/kg xylanase	30%	21-day feeding	Increased BW, ADG, AID of GE, and NDF of the pigs Decreased viscosity of jejunal digesta, TNF-α, and PYY in the plasma of the pigs	[112]
Barrows 30.4 ± 2.20 kg	600 FTU/kg phytase	10%	28-day feeding	Increased ADG of the pigs	[118]
Barrows 30.4 ± 2.20 kg	0.5 g/kg multi-carbohydrase	10%	28-day feeding	Increased ADG and ADFI of the pigs	[118]
Barrows 22.4 ± 1.40 kg	600 FTU/kg phytase	10%	28-day feeding	Increased ASH, P, and Ca in total tract digestibility of nutrients, urinary P, and P retained in the pigs Decreased fecal P and total P of the pigs	[118]
Barrows 22.4 ± 1.40 kg	0.5 g/kg multi-carbohydrase	10%	28-day feeding	Increased ASH, P, and Ca in total tract digestibility of nutrients, urinary P, and P retained in the pigs Decreased Fecal P and Total P of the pigs	[118]
Pigs 57.6 kg	200, 400, 600 U/kg mannanase	10%	28-day feeding	Increased ADG, blood glucose, ATTD of DM, and CP of the pigs	[114]
Pigs 92.7 kg	200, 400, 600 U/kg mannanase	10%	28-day feeding	Increased ADG, blood glucose, ATTD of DM, GE, and CP of the pigs	[114]
Pigs 60.5 kg	400 U/kg mannanase	15%	23-day feeding	Increased ADG and blood glucose in the pigs	[114]
Pigs 86.5 kg	400 U/kg mannanase	15%	29-day feeding	Increased ADG and blood glucose, ATTD of DM, GE, and CP of the pigs	[114]

MCT1, monocarboxylate transporter 1; AID, apparent ileal digestibility; GE, gross energy; BW, body weight; ADG, average daily gain; NDF, neutral detergent fiber; TNF-α, tumor necrosis factor-alpha; PYY, peptide YY; ADFI, average daily feed intake; P, phosphorus; Ca, calcium; and ATTD, apparent total tract digestibility.

### 3.2. Pretreatment

DDGS offers rich caloric energy and digestible protein in pig feed rations [119]. However, the high-fiber content of DDGS can curtail pig intake and compromise the digestibility of energy and other nutrients when incorporated into pig diets, thereby impacting its production performance [120]. To mitigate these challenges, pretreatment methods can be employed to enhance the nutritional quality of DDGS. We summarize the impact of various pretreatment techniques on the nutritional value of DDGS in pig feed, considering the different feed processing methods (Table 7 and Table 8).

Numerous studies highlight the efficacy of fermentation in improving the nutrient utilization and growth performance of the pigs on diets supplemented with DDGS (Table 7). The incorporation of fermentation enzymes into the DDGS production process facilitates NSP degradation via their inherent liquid fermentation mechanisms [121]. Jakobsen et al. [121] corroborated this finding, noting that the liquid fermentation of DDGS increased the ATTD of DM, CP, P, and total NSP ATTD of the pigs compared to those fed unfermented DDGS, whereas Rho et al. [122] demonstrated that fermented DDGS with exogenous fiber-degrading enzymes increased the ATTD of CF in the pigs compared to those fed unfermented DDGS. In addition, Wiseman et al. [123] determined that diets of DDGS fermented with β-glucanase and xylanase improved the growth performance of the pigs compared to those fed unfermented DDGS. Rho et al. [17] also observed that the liquid fermentation of DDGS with β-glucanase and xylanase increased the intestinal crypt depth of the pigs compared to those fed unfermented DDGS. Moran et al. [124] found that DDGS fermented with xylanase increased the ATTD of GE and jejunal crypt depth of the pigs compared to those fed unfermented DDGS. The findings from these studies collectively suggest that fermentation enhances the feed utilization of DDGS in pigs, which subsequently leads to improved growth performance and intestinal health in growing and finishing pigs.

**Table 7:** The effect of feeding fermented corn distillers dried grains with solubles (DDGS) on pigs.

Animal	Fermented Study		Feeding Study			Reference
	Enzyme	Treatment Conditions	DDGS Levels	Experiment Duration	Main Effects	
Pigs 25 ± 1.0 kg	β-glucanase and xylanases	3–10 d at 40 °C	30%	42-day feeding	Increased feed efficiency in the first three weeks of feeding and ATTD of DM, CP, GE, and crypt depth of the pigs	[17]
Barrows 35.1 ± 1.8 kg	mixture of xylanase and glucanase	6 d at 20 °C	60%	4 × 14-day feeding	Increased ATTD of DM, CP, P, total NSPs, AID of total NSP, and digestibility of DDGS in the pigs Decreased ileal butyric acid levels of the pigs	[121]
Barrows 35.1 ± 1.8 kg	mixture of cellulase and xylanase	6 d at 20 °C;	60%	4 × 14-day feeding	Increased ileal LAB levels, ATTD of DM, CP, P, total NSPs, AID of total NSPs of the pigs Decreased ileal butyric acid levels in the pigs	[121]
Pigs 20 kg	Fiber-degrading enzymes	24 h at 40 °C	65%	3 × 11-day feeding	Increased ATTD of CF in the pigs	[122]
Pigs 5.9 ± 0.6	β-glucanase and xylanase	5–12 d at 40 °C	7.5, 25%	34-day feeding	Increased growth performance of the pigs	[123]
Pigs 5.9 ± 0.6	silage inoculant biotal plus	1–7 d at 40 °C	7.5, 16.5, 25%	34-day feeding	Increased ADG, and BW of the pigs and favors the growth of the low-weight weaned piglets during extended nursery period	[123]
Pigs 25.87 ± 0.38 kg	xylanase	24 h at 22 °C	30%	16-day feeding	Increased ATTD of GE and jejunal crypt depth of the pigs	[124]
Pigs 25.87 ± 0.38 kg	xylanase	24 h at 22 °C	30%	16-day feeding	Increased AID of NDF, N, jejunal villus height, and crypt depth of the pigs	[124]

ATTD, apparent total tract digestibility; DM, dry matter; CP, crude protein; GE, gross energy; NSPs, non-starch polysaccharides; AID, apparent ileal digestibility; CF, crude fiber; ADG, average daily gain; BW, body weight; N, nitrogen; SID, standardized ileal digestible; NDF, neutral detergent fiber; and LAB, lactic acid bacteria.

A number of processing techniques other than fermentation have been developed that can minimize production costs while achieving the highest growth performance [125] (Table 8). Digestion and absorption are pivotal processes as they control the absorption of nutrients and energy from the diet. The pelletizing of pig diets involves a feed processing technique in which the diet is subjected to heat or moisture and subsequently pressed through a mold to amalgamate smaller particles into a cohesive, larger unit [126]. Research by Overholt et al. [20] indicated that the addition of diets pellets containing DDGS to the diet led to increased hot carcass weight (HCW) and carcass fat at slaughter of growing and finishing pigs compared to those fed diets without pelleting containing DDGS. Feeding pellets has been demonstrated to enhance nutrient digestibility and feed efficiency relative to feeding DDGS without pelleting [127]. However, both HM and RM techniques can enhance the digestive process by reducing the particle size of the feed ingredients, thereby optimizing feed utilization [21]. Research by Acosta et al. [21] highlighted that the use of HM or RM DDGS enhanced the ATTD of the DM, GE, acid hydrolyzed ether extract, and N in the pigs compared to those fed without HM or RM DDGS. Extruded DDGS exhibits enhanced nutrient utilization and a reduced amount of antinutritional factors [126]. Zhang et al. [18] highlighted that the extrusion of DDGS notably elevated both digestive and metabolic energy, which led to a significant improvement in ATTD values for energy, DM, organic matter, and NDF in the pigs compared to those fed DDGS without extrusion. Changing the particle size of feedstuffs during pelleting, HM and RM, and extrusion changes the physicochemical characteristics of the structure of grain particle, thus improving nutrient utilization of the pigs [128]. In addition, this process of sieving and elutriation effectively reduces the fiber content in the raw material, which results in a total dietary fiber concentration such that it is roughly 3.5% lower than that of traditional DDGS, decreasing it from 28.7% to 25.2% [129]. Research by Soares et al. [19] indicated that the sieving and elutriation of DDGS enhanced the digestive energy and the values of ME and NE in growing and finishing pigs when compared to those fed DDGS without sieving and elutriation.

**Table 8:** The effect of feeding pretreated corn distillers dried grains with solubles (DDGS) on pigs.

Animal	Pretreatment Method	DDGS Levels	Experiment Duration	Main Effects	Reference
Pigs 36.0 ± 1.8 kg	Extrusion	29.07%	12-day feeding	Increased DE, ME, ATTD of the GE, DM, and NDF of the pigs A tendency to increase ATTD of CP and ADF of the pigs	[18]
Pigs 20.3 ± 1.8 kg	Extrusion	29.07%	21-day feeding	Increased AID of total indispensable amino acids of the pigs	[18]
Barrows 23.0 kg ± 2.8 kg	Sieving and elutriating	40%	14-day feeding	Increased DE of the pigs	[19]
Barrows 73.0 ± 1.8 kg	Sieving and elutriating	40%	14-day feeding	Increased DE and ME of the pigs	[19]
Pigs 25.75 ± 2.29 kg	Pelleting	30%	91-day feeding	Increased growth performance, HCW, carcass fat, and slaughter of the pigs	[20]
Pigs 11.77 ± 0.12 kg	Pelleting	30%	21-day feeding	Increased ATTD of DM, OM, energy, CP, fat, NDF, and ADF in the pigs	[127]
Pigs 18.40 ± 0.18 kg	Pelleting	30%	14-day feeding	Increased ADG and G:F of the pigs	[127]
Pigs 54.7 ± 0.9 kg	Hammermill or roller mill	45%	11-day feeding	Increased the ATTD of DM, GE, and AEE of the pigs A tendency to increase the ATTD of N of the pigs	[21]
Barrows 55.2 ± 3.6 kg	Cold fermented	50%	7-day feeding	Increased SID of amino acids in the pigs	[70]

DE, digestible energy; ME, metabolizable energy; ATTD, apparent total tract digestibility; GE, gross energy; NDF, neutral detergent fiber; CP, crude protein; ADF, aid detergent fiber; AID, apparent ileal digestibility; HCW, hot carcass weight; G:F, gain to feed; AEE, acid hydrolyzed ether extract; and N, nitrogen.

### 3.3. Metabolic Regulation

DDGS contains substantial amounts of PUFAs [53], which has the potential to adversely influence the growth performance and carcass characteristics of growing and finishing pigs [130]. However, metabolic regulation via adding functional nutrients to the diets of pigs can effectively mitigate these drawbacks to enhance the overall feeding value of DDGS (Table 9).

Conjugated linoleic acid (CLA) is an important functional nutrient that consists of a group of positional and geometric (cis or trans) isomers of linoleic acid with conjugated double bonds [130]. In the study by Pompeu et al. [92], it was found that the addition of 0.6% CLA to diets containing DDGS increased the ADG, gain-to-feed ratio (G:F), carcass gain, and lean and SFAs of the pigs compared to those fed DDGS alone and decreased the carcass dressing, C18:1n9, MUFAs, and iodine value (IV) of the pigs compared to those fed DDGS alone. However, in the study by Wang et al. [131], supplementation of diets containing DDGS with 1% CLA increased the proportions of C18:0 for backfat and C14:0 and C16:0 for belly fat of the pigs compared to those fed DDGS alone and decreased the proportions of C18:2, C18:3, C20:1, C20:3, and PUFAs for backfat and C18:2 and PUFAs for belly fat in the pigs compared to those fed DDGS alone. Adding CLA to diets containing DDGS increased stearic acid and decreased linoleic acid in the pigs compared to those fed DDGS alone, which suggests changes in fatty acid metabolism at the adipose tissue level. Previous studies have shown that CLA can reduce SCD-1 and the ∆9 desaturase index in pigs [132]. Smith et al. [132] concluded that, although the ∆9 desaturase index is not a direct indicator of absolute SCD-1 enzyme activity, it is associated with changes in SCD-1 enzyme activity, whereas in the study by White et al. [130], it was found that SCD-1 mRNA expression was not reduced in the adipose tissue of the pigs fed a CLA diet containing DDGS compared to those fed DDGS alone, and the ∆9 desaturase index was reduced. This decrease in ∆9 desaturase index indicates a response of SCD-1 activity to CLA feeding, which coincides with a decrease in SCD-1 mRNA expression [132].

Similarly, to address the effects of high linoleic acid content in DDGS, such as shortening the shelf life of pork, Wang et al. [131] reported that the addition of 1 g/kg betaine to DDGS increased the SFA content and decreased the C18:2 and PUFA content of the pigs compared to those fed DDGS alone. Betaine is a nontoxic amino acid derivative that is widely distributed in nature [131]. L-carnitine plays an important role in lipid catabolism by transferring long-chain fatty acids into the mitochondrial matrix for fatty acid β-oxidation [133]. Ying et al. [27] reported that the C18:2n-6 and C20:2 contents of the pigs decreased when L-carnitine was added to diets containing 30% DDGS compared to those fed diets containing only 30% DDGS. However, in the study by Meng et al. [54], it was found that L-carnitine supplementation of diets supplemented with 80% DDGS did not change the fatty acid composition of the pigs. It is possible that in the experiments of Ying et al. [27] the diets supplemented with 30% DDGS, the lecithin of soybean meal interacted with L-carnitine to reduce the C18:2n-6 and C20:2 contents of the pigs. Ractopamine is a phenethanolamine with similar properties to β-adrenergic agonists and works by directing nutrients from fat deposition toward protein accretion [134]. In an experiment by Pompeu et al. [92], adding 7.4 mg/kg ractopamine to diets containing DDGS increased the ADG, G:F, HCW, loin depth, lean muscle, C16:1, C18:2n6, MUFAs, PUFAs, total omega-6, and IV and decreased the ADFI, back fat depth, and SFAs of the pigs compared to those fed DDGS alone. CLA, betaine, and L-carnitine can reduce the effects of feeding DDGS, but it is worthwhile to investigate how to increase the shelf life of pork after feeding DDGS.

Vitamin E (a-tocopherol) is a membrane-associated antioxidant that can maintain cellular integrity and effectively delay lipid oxidation. In the experiments of Wang et al. [68], it was found that the concentration of the total volatile basic nitrogen (TVB-N) and thiobarbituric acid reactive substances (TBARS) in pork was decreased by the supplementation of vitamin E in diets containing DDGS compared to those fed diets containing only DDGS. TVB-N concentration is an important freshness index of meat, and the TBARS value is a primary indicator of lipid oxidation in meat products during storage [135,136]. This suggests that the addition of vitamin E to DDGS can extend the shelf life of pork. In study by Xu et al. [137], it was observed that supplementation of vitamin E improved lipid oxidative stability via increasing vitamin E retention, rather than the regulation by gene expression of the MAPK–Nrf2 signaling pathway. Collectively, these functional nutrients address the inherent limitations of DDGS in practical production. When incorporated into diets containing DDGS utilization strategies, they enhance their nutritional profile and mitigate the environmental challenges associated with DDGS disposal.

**Table 9:** The effect of supplementation with functional nutrients on pigs fed corn distillers dried grains with solubles (DDGS).

Animal	Improvement Mode	DDGS Levels	Experiment Duration	Main Effects	Reference
Barrows 100.4 ± 3.7 kg	0.6% CLA	20%	27-day feeding	Increased the ADG, G:F, carcass gain, lean muscle, and SFAs of the pigs Decreased the carcass dressing, C18:1n9, MUFAs, and IV of the pigs	[92]
Barrows 96 ± 1.38 kg	0.6% CLA	20, 40%	Final BW 105 ± 1.75 kg	Decreased the ∆9 desaturase index in adipose tissue and outer layer backfat IV of the pigs	[130]
Barrows 60 ± 2 kg	10 g/kg CLA	30%	42-day feeding	Increased the proportions of C18:0 and SFAs for back fat and C14:0, C16:0, and SFAs for belly fat of the pigs Decreased the proportions of C18:2, C18:3, C20:1, C20:3, PUFAs, and IV for backfat and C18:2, PUFAs, and IV for belly fat of the pigs	[131]
Barrows 60 ± 2 kg	1 g/kg betaine	30%	42-day feeding	Increased the proportions of C16:0, C18:0, C22:0, and SFAs for backfat and C14:0, C16:0, and SFAs for belly fat of the pigs Decreased the proportions of C18:2, PUFAs, and IV for backfat and C18:2, PUFAs, and IV for belly fat of the pigs	[131]
Barrows 100.4 ± 3.7 kg	7.4 mg/kg ractopamine	20%	27-day feeding	Increased the ADG, G:F, HCW, carcass dressing, carcass gain, carcass G:F, loin depth, lean muscle, C16:1, C18:2n6, MUFAs, PUFAs, total omega-6 and IV of the pigs Decreased ADFI, back fat depth, and SFAs of the pigs	[92]
Pigs 58 ± 2 kg	10, 210 IU/kg vitamin E	15, 30%	42-day feeding	Increased a-tocopherol concentrations in the plasma, liver, muscle, and adipose tissue of the pigs Decreased shear and drip losses and the proportion of SFAs in abdominal fat, subcutaneous fat, and intramuscular fat of the pigs	[68]
Barrows 7 ± 0.3 kg	11, 110 IU/kg vitamin E	30%	Final BW 50 kg	Increased the liver GSH concentration and serum GPx activity of the pigs	[138]
Pigs 6.6 ± 0.4 kg	0.032, 0.32% vitamin E	30%	Final BW 107 kg	No beneficial effects on the pigs	[139]
Barrows 45±1.7kg	50 mg/kg L-carnitine	80%	80-day feeding	Increased a* of pork and CPT1A, HSL, FABP4, CRAT of the pigs Decreased the backfat thickness and FAS of the pigs	[54]
Barrows 36 ± 1 kg	50, 100 mg/kg L-carnitine	20, 30%	109-day feeding	Increased the HCW, greater carcass yields, greater fat depths, purge loss, and fresh LM color scores of the pigs Decreased the C18:2n-6 and C20:2 contents of the pigs	[27]

ADG, average daily gain; G:F, gain to feed; SFAs, saturated fatty acids; MUFAs, monounsaturated fatty acids; IV, iodine value; HCW, hot carcass weight; PUFAs, polyunsaturated fatty acids; ADFI, average daily feed intake; GSH, glutathione; GPx, glutathione peroxidase; LM, longissimus muscle; BW, body weight; ACCα, acetyl-CoA carboxylase; ME1, malic enzyme 1; CPT1A, carnitine palmitoyltransferase 1A; HSL, hormone-sensitive lipase; FABP4, fatty acid binding protein 4; CRAT, carnitine acetyltransferase; FAS, fatty acid synthase; IMF, intramuscular fat; SCD, stearoyl-CoA desaturase; a*, redness index.

### 3.4. Oils

DDGS contains substantial amounts of PUFAs [53], which can lead to problems such as softening of pork fat and decrease the overall quality of pork carcasses [53,130]. Thus, many studies have utilized oils and by-product from oils, including tallow, cottonseed oil, soybean oil (SBO), and crude glycerol to address the issues of balanced fatty acid composition and carcass quality to promote better utilization of DDGS (Table 10).

Tallow is a readily available, economical source of fat with a relatively high SFA content, which can improve the fatty acid composition of the pigs fed diets containing DDGS [140]. In a study by Coble et al. [141], it was observed that adding 5% tallow to diets containing DDGS during the final three weeks before slaughter enhanced the growth performance of the pigs compared with those fed a diet of DDGS alone but did not mitigate the adverse effects of high-fiber diets on the carcass yield of the pigs compared to those fed DDGS alone. However, in experiments by Davis et al. [67,140], they investigated the supplementation of 5% tallow to diets containing DDGS and observed an increase in G:F and carcass yield of the pigs and a decrease in the AID of C18:0, SFAs, ATTD of C16:0, IV of belly fat, and ADFI in growing pigs compared with those fed DDGS alone. Furthermore, adding 10% tallow to diets containing DDGS led to an increased AID of MUFAs and the ATTD of C18:0 and decreased the AID of PUFAs in the pigs compared to those fed DDGS alone [140]. 

Cottonseed oil is an easily accessible vegetable oil source for livestock production, rich in cyclopropene fatty acids that are known to inhibit desaturase enzymes responsible for synthesizing unsaturated fatty acids in the body [142,143]. The addition of 5% refined cottonseed oil to diets containing DDGS improved the pig growth performance, carcass characteristics, and abdominal fat melting point of the pigs compared to those fed a diet containing DDGS alone [144]. The lack of improvement in pork fat hardness may have been caused by the removal of cyclopropene fatty acids from the cottonseed oil during treatment by the authors [145]. 

SBO, as one of the most consumed vegetable oils worldwide, is rich in polyunsaturated fatty acids and is used to increase the absorbable concentration of dietary energy and acid hydrolyzed ether extract (AEE) digestibility [146,147]. Gutierrez et al. [69] found that 6% SBO-supplemented DDGS diets increased the AID and ATTD of AEE and decreased the AID and ATTD of NDF in the pigs compared with those fed DDGS supplemented with 2% SBO. 

Crude glycerol is a by-product formed during the trans-esterification reaction among oil, methanol, and a catalyst [148]. It is rich in glycerol, ash, and methanol, which may affect the cellular metabolism involved in lipid synthesis, reduce the linoleic acid content and, consequently, the PUFA content of the pigs fed diets containing DDGS [148,149,150,151]. Adding concentrations of 2.5%, 5%, or 10% crude glycerol to DDGS diets led to a significant increase in MUFAs in the pigs compared with those fed DDGS alone [26,152]. Notably, adding 10% crude glycerol to pig diets containing DDGS resulted in a marked decrease in PUFAs compared with feeding DDGS alone. However, in the study by Villela et al. [144], it was observed that 8% crude glycerol in pig diets containing DDGS had no discernible impact on the proportion of fatty acids and growth performance of the pigs compared with those fed DDGS alone. This difference in results may be because of the different chemical compositions of the crude glycerol [144].

Collectively, these strategies highlight the potential of tailored lipid supplementation to modulate fatty acid profiles and enhance DDGS utilization in pig diets, though outcomes depend on oil type, inclusion level, and processing methods. Further research is warranted to standardize by-product compositions and optimize dietary formulations for consistent carcass quality improvements.

**Table 10:** The effect of adding oils on pigs fed corn distillers dried grains with solubles (DDGS).

Animal	Improvement Mode	DDGS Levels	Experiment Duration	Main Effects	Reference
Pigs 25 kg	5%, 10% tallow	30%	10-day feeding	Increased AID of SFAs, ileal digestibility of C16:0, C18:0, C18:1, and SFAs, and ATTD of MUFAs in the pigs Decreased AID of C18:0 and SFAs and ATTD of C16:0 of the pigs	[140]
Pigs 32.4 ± 1.9 kg	5% tallow	30%	Final BW 113 kg	Increased G:F and carcass yield of the pigs Decreased IV of belly fat and ADFI of the pigs	[67]
Pigs 105.8 ± 0.1 kg	5% tallow	30%	20-day feeding	Increased ADG and G:F of the pigs	[141]
Barrows 31.0 ± 1.1 kg	2.5%, 5% crude glycerol	20%	97-day feeding	Increased myristic acid and MUFAs in the jowl fat and backfat of the pigs	[26]
Pigs 36.5 ± 0.5 kg	10% crude glycerin	15%, 25%	84-day feeding	Increased MUFAs and C18:1 of the pigs Decreased PUFAs and C18:2 of the pigs	[152]
Pigs 24 ± 4 kg	5% minimally refined cottonseed oil or 8% crude glycerol	40%	Final BW 115 ± 8 kg	Increased ADG, G:F, final BW, HCW, and melting point of the belly fat in the pigs	[144]
Pigs 33.8 ± 2.2 kg	2%, 6% SBO	20%, 40%	52-day feeding	Increased AID and ATTD of AEE of the pigs Decreased AID and ATTD of NDF of the pigs	[69]

monounsaturated fatty acids (MUFAs), apparent ileal digestibility (AID), apparent total tract digestibility (ATTD), neutral detergent fiber (NDF), average daily gain (ADG), gain to feed (G:F), saturated fatty acids (SFAs), average daily feed intake (ADFI), body weight (BW), hot carcass weight (HCW), polyunsaturated fatty acids (PUFAs), and acid hydrolyzed ether extract (AEE).

### 3.5. Optimal Amino Acid Content

DDGS contains zein, which contains a large amount of Leu; thus, the addition of a large amount of DDGS to the diet will generate an excess of Leu in the diet. Leu, along with Val and Ile, are branched-chain amino acids (BCAAs), which share identical enzymatic steps in their preceding catabolic processes [30]. Because of these shared catabolic pathways, an overabundance of any BCAA elevates the breakdown of all BCAAs, which subsequently results in an amino acid deficiency [31]. Amino acid deficiency may result in insufficient protein deposition and, consequently, a reduction in the growth performance of the pigs [28,153,154]. It has been shown that high levels of Val, Ile, and Trp during the growth period of pigs mitigated the negative effects of excessive dietary Leu [155]. Therefore, researchers accurately estimate the optimal ratios of Val, Ile, and Trp to Leu by experimentally determining the growth performance of the pigs when fed diets containing high amounts of DDGS (Table 11).

Clizer et al. [156] reported that the G:F response was greatest when a SID Val:Lys ratio of 70% was provided from day 0–14, and from day 14–28 d and during the cumulative period, when the SID Val:Lys ratio was 75%. The overall growth performance from day 0–28 peaked at a SID Val:Lys ratio of 75%. However, the NRC [128] recommendation of a SID Val:Lys ratio of 65.3% for 25–50 kg pigs was insufficient for the 40–70 kg pig model used by Clizer et al. [128,156]. This is because the Lys ratios determined by the study of dietary amino acids required in pigs between 25 and 75 kg in the NRC [128] were conducted on pigs with a final BW of 33 kg [128]. As pigs grow and their BW and intestinal tissue size expand, their amino acid requirements correspondingly increase [157]. Whereas as the proportion of DDGS in the diets increases, the concentration of Ile in the diet decreases more rapidly than Val, which can lead to Ile becoming the first limiting amino acid in DDGS-containing diets earlier than Val [153,154,156]. In the study by Clizer et al. [153], pigs at the 82–100 kg stage and 100–130 kg stage exhibited the best growth performance when the SID Ile:Lys ratio was 70%. However, the NRC [128] estimated the SID Ile:Lys ratio requirements at 53.4% for 75–100 kg pigs and 54.1% for 100–135 kg pigs [128]. The reason for this may be because the feeding of a DDGS-containing diet to pigs leads to changes in their amino acid requirements [28].

Kwon et al. [158] reported that excess Leu in the diet reduces the brain uptake of Trp. However, an increase in Trp content may mitigate the negative effects of Leu on ADFI in pigs by reducing the transport of Leu across the blood–brain barrier [159]. In a study by Clizer et al. [28], it was found that optimal growth performance and carcass characteristics were observed when the SID Trp:Lys ratio was 24% in a diet containing 40% DDGS. The reduced ADG response to an increased SID Trp:Lys ratio in the diet during the fattening period may indicate that the dietary Lys content is excessive. We can judge the optimal addition of Lys by observing the ADG response to an increase in the SID Trp:Lys ratio [160]. Therefore, if lower concentrations of Lys are added to fattening diets, different responses may be observed. Salyer et al. [161] reported that optimal growth performance was observed in 36.3 kg pigs fed diets containing 30% DDGS for days 0–42 with a 16% SID Trp:Lys ratio, whereas optimal growth performance was observed in 73.3 kg pigs fed diets containing 30% DDGS for days 42–105 with a SID Trp:Lys ratio of 18%. In another experiment, optimal growth performance was observed in 66.3 kg pigs fed diets containing 30% DDGS for days 0–73 with a SID Trp:Lys ratio of 19.5% [161]. However, the NRC (2012) recommends an optimal SID Trp:Lys ratio of 17.4% for growing and finishing pigs [128]. In these experiments, pigs fed DDGS-containing diets had higher optimal SID amino acid:Lys ratios those that of the NRC [128]; therefore, amino acid requirements need to be redefined for precision nutrition when feeding DDGS diets.

Because DDGS contains a high content of insoluble fiber, feeding diets containing DDGS affect pig gastrointestinal epithelial cells, digestive enzymes, and mucosal secretions, of which Thr is a major component [162,163]. However, it has been shown that increasing the SID Thr:Lys ratio in high-fiber diets increased the growth performance of the pigs when compared to those fed low-fiber diets [164]. However, in the study of Tolosa et al. [165], it was found that increasing the SID Thr:Lys ratio did not affect the growth performance of the pigs.

In summary, the inclusion of high levels of DDGS in pig diets introduces excessive Leu, disrupting BCAA metabolic equilibrium and subsequently impairing growth performance. Current research indicates that optimizing the ratios of Val, Ile, and Trp relative to Leu—such as standardized ileal digestible (SID) Val:Lys at 75%, Ile:Lys at 70%, and Trp:Lys between 19.5% and 24%—can mitigate the adverse effects of excess Leu. However, these requirements substantially exceed the recommendations outlined by the NRC [128], suggesting that conventional amino acid standards may not align with high-DDGS dietary systems. Additionally, while the high insoluble fiber content in DDGS may alter Thr demand by modulating gastrointestinal function, findings on Thr supplementation remain inconsistent. Future studies should prioritize systematically quantifying precise amino acid ratios tailored to distinct growth phases, DDGS inclusion rates, and production objectives. Concurrently, elucidating the interplay between fiber and amino acid metabolism is critical to refining dynamic nutritional models that account for ingredient-specific characteristics, thereby advancing the strategic utilization of DDGS in pig nutrition.

**Table 11:** The effect of optimal amino acids on pigs fed corn distillers dried grains with solubles (DDGS).

Animal	Improvement Mode	DDGS Levels	Experiment Duration	Main Effects	Reference
Pigs 35.1 ± 0.5 kg	SID Thr:Lys Formulated in four phases 61, 62, 63, 65% vs. 67, 68, 69, 72%	40%	112-day feeding	No beneficial effects on the pigs	[165]
Pigs 39.4 kg	SID Val:Lys 60%, 65%, 70%, 75%, 80%	30%	28-day feeding	Increased growth performance of the pigs	[156]
Pigs 82.3 ± 0.39 kg	SID Ile:Lys 55%, 60%, 65%, 70%, 75%	20%	56-day feeding	Increased loin depth and tendency to increase the lean muscle percentage of the pigs Decreased back fat of the pigs	[153]
Pigs 36.3 kg	SID Trp:Lys 14.0, 15.0, 16.5, 18.0%	40%	105-day feeding	Increased final BW, ADG, G:F, and HCW of the pigs	[161]
Pigs 66.3 kg	SID Trp:Lys 15.0, 16.5, 18.0, 19.5%	40%	73-day feeding	Increased final BW, ADG, G:F, and HCW of the pigs Decreased FFLI of the pigs with added crystalline Trp	[161]
Pigs 38.6 ± 0.2 kg	SID Trp:Lys 15, 18, 21, 24%	30%	98-day feeding	Increased ADG, ADFI, final BW, and carcass hot weight of the pigs	[28]

BW, body weight; ADG, average daily gain; G:F, gain to feed; HCW, hot carcass weight; and FFLI, fat-free lean index.

## 4. Conclusions

Corn DDGS offers a cost-effective and sustainable protein source for pig feed, providing economic and environmental benefits when replacing 20–30% of soybean meal while maintaining growth performance and carcass quality. Despite its advantages, practical implementation faces challenges from high fiber content, inconsistent nutrient composition, and PUFAs affecting fat stability. Current mitigation strategies—including enzyme additives, pretreatment techniques, and lipid supplementation—show partial success, though batch-to-batch quality variations across regions remain a major limitation. To optimize DDGS utilization, priority should be given to developing region-specific quality grading systems incorporating fiber, amino acid, and PUFA metrics rather than fat content alone, establishing predictive databases linking corn genetics and ethanol processing parameters to nutritional profiles, and refining synergistic technologies like enzymatic pretreatment with low-temperature drying to preserve nutrients. Expanding applications through functional peptide extraction or microbial protein production could extend its value beyond conventional feed uses, while lifecycle assessments and IoT-enabled ethanol production monitoring would, respectively, quantify environmental benefits and ensure quality consistency. Addressing these priorities could transform DDGS from an industrial by-product into a pillar of sustainable pork production systems.

## Figures and Tables

**Table 1:** Nutrient variability in corn distillers dried grains with solubles (DDGS).

Item	Olukosi and Adebiyi [41]			Cromwell et al. [39]			Spiehs et al. [43]			Belyea et al. [42]		
	Mean	Range	CV	Mean	Range	CV	Mean	Range	CV	Mean	Range	CV
DM, %	ND	ND	ND	90.5	87.1–92.7	1.8	88.9	87.2–90.2	1.7	ND	ND	ND
Starch, %	ND	ND	ND	ND	ND	ND	ND	ND	ND	5.3	4.7–5.9	9.7
Total carbohydrates, %	ND	ND	ND	54.3	ND	ND	53.1	ND	ND	52.1	ND	5.2
CF, %	7.4	6.2–11.3	15.1	ND	ND	ND	8.8	8.3–9.7	8.7	10.2	9.6–10.6	3.7
ADF, %	13.6	8.6–18.5	24.2	15.9	11.4–20.8	21.1	16.2	13.8–18.5	28.4	16.8	15.4–19.3	9.3
NDF, %	36.6	27.7–51.0	15.7	38.8	33.1–43.9	10.0	42.1	36.7–49.1	ND	14.3	ND	ND

ND, no data; DM, dry matter; CF, crude fiber; ADF, aid detergent fiber; NDF, neutral detergent fiber; and CV, coefficient of variation.

**Table 2:** Fatty acid composition of corn distillers dried grains with solubles (DDGS) based on feedstock grain.

Item	Mean	SEM	Wu et al. [52]	Ma et al. [53]	Meng et al. [54]	Meng et al. [55]
EE, %	7.88	1.1678	10.7	6.73	5.36	8.74
C14:0, %	0.20	0.0650	0.13	0.26	ND	ND
C16:0, %	12.23	1.3855	15.70	8.99	11.63	12.60
C16:1, %	0.16	0.0316	0.20	0.22	0.08	0.14
C18:0, %	2.59	0.2370	2.19	3.03	2.97	2.17
C18:1n-9, %	23.97	1.2465	24.27	21.51	22.78	27.30
C18:2n-6, %	52.97	1.2590	53.53	49.88	55.96	52.50
C18:3n-3, %	2.11	0.9633	1.80	0.37	4.85	1.40
Total SFAs, %	15.99	1.0833	19.11	14.14	15.11	15.60
Total MUFAs, %	24.77	0.9453	24.84	23.67	23.16	27.40
Total PUFAs, %	55.66	2.0409	55.32	51.40	61.21	54.70

EE, ether extract; SFAs, saturated fatty acids; MUFAs, monounsaturated fatty acids; and PUFAs, polyunsaturated fatty acids.

**Table 4:** Apparent ileal digestibility and standardized ileal digestibility of indispensable and dispensable amino acids in corn distillers dried grains with solubles (DDGS) by pigs.

Item	DDGS								
	Mean	SEM	Max	Min		Mean	SEM	Max	Min
Indispensable AA AID					Indispensable AA SID				
Arg, %	79.28	1.67	85.00	68.80		84.79	1.6531	89.30	79.10
His, %	76.16	1.86	83.10	68.60		77.59	1.9077	85.70	71.90
Ile, %	75.43	1.62	82.50	70.20		77.37	1.4601	84.40	73.60
Leu, %	83.26	1.35	88.60	76.00		84.34	1.6249	90.80	77.30
Lys, %	62.50	6.40	85.20	27.60		63.94	4.4456	86.20	50.40
Met, %	82.68	1.78	90.00	74.90		82.80	1.5934	88.80	76.50
Phe, %	81.83	2.31	97.30	74.00		82.17	1.2972	87.50	76.90
Thr, %	67.29	1.89	75.40	60.20		72.34	1.6771	78.10	66.70
Trp, %	72.81	3.57	86.40	55.10		71.67	2.2305	78.00	62.40
Val, %	74.42	2.33	87.90	66.10		75.99	1.4255	82.30	70.50
Dispensable AA AID					Dispensable AA SID				
Ala, %	74.72	1.55	87.90	66.10		80.91	1.6658	86.60	74.60
Asp, %	65.00	1.17	72.70	60.90		73.04	1.9196	78.78	65.40
Cys, %	64.67	2.74	75.30	46.90		73.86	1.9582	79.90	67.30
Glu, %	78.01	1.26	84.40	72.20		82.80	1.7456	88.28	76.50
Gly, %	47.24	3.65	68.10	34.67		71.10	2.8112	88.90	58.70
Pro, %	52.28	6.55	79.20	33.70		82.81	3.3131	95.90	71.60
Ser, %	72.29	1.40	79.00	68.00		79.73	2.0133	83.85	73.50
Tyr, %	82.35	1.88	92.60	75.60		88.01	1.7686	94.20	81.90

AA, amino acid; SID, standardized ileal digestible; Max, maximum; Min, minimum; and SEM, standard error of the mean. Compiled from [18,69,70,71,77,78,79,80,81,82].

## Data Availability

Not applicable.
